# The Therapeutic Effects and Mechanisms of Quercetin on Metabolic Diseases: Pharmacological Data and Clinical Evidence

**DOI:** 10.1155/2021/6678662

**Published:** 2021-06-23

**Authors:** Huan Yi, Hengyang Peng, Xinyue Wu, Xinmei Xu, Tingting Kuang, Jing Zhang, Leilei Du, Gang Fan

**Affiliations:** ^1^State Key Laboratory of Southwestern Chinese Medicine Resources, School of Pharmacy, Chengdu University of Traditional Chinese Medicine, Chengdu 611137, China; ^2^Animal Health Research Institute, Tongwei Co., Ltd., Chengdu 610041, China; ^3^School of Ethnic Medicine, Chengdu University of Traditional Chinese Medicine, Chengdu 611137, China

## Abstract

Metabolic diseases have become major public health issues worldwide. Searching for effective drugs for treating metabolic diseases from natural compounds has attracted increasing attention. Quercetin, an important natural flavonoid, is extensively present in fruits, vegetables, and medicinal plants. Due to its potentially beneficial effects on human health, quercetin has become the focus of medicinal attention. In this review, we provide a timely and comprehensive summary of the pharmacological advances and clinical data of quercetin in the treatment of three metabolic diseases, including diabetes, hyperlipidemia, and nonalcoholic fatty liver disease (NAFLD). Accumulating evidences obtained from animal experiments prove that quercetin has beneficial effects on these three diseases. It can promote insulin secretion, improve insulin resistance, lower blood lipid levels, inhibit inflammation and oxidative stress, alleviate hepatic lipid accumulation, and regulate gut microbiota disorders in animal models. However, human clinical studies on the effects of quercetin in diabetes, hyperlipidemia, and NAFLD remain scarce. More clinical trials with larger sample sizes and longer trial durations are needed to verify its true effectiveness in human subjects. Moreover, another important issue that needs to be resolved in future research is to improve the bioavailability of quercetin. This review may provide valuable information for the basic research, drug development, and clinical application of quercetin in the treatment of metabolic diseases.

## 1. Introduction

Metabolic diseases encompass a constellation of maladies characterized by disruption of normal metabolism, involving the processing or transport of proteins (amino acids), carbohydrates (sugars and starches), or lipids (fatty acids) [1]. Diabetes is a kind of common endocrine metabolic diseases. According to the latest report from the International Diabetes Federation, about 463 million adults (20-79 years old) worldwide were suffering from diabetes, and the number of diabetic patients in China ranked first [2]. It is reported that metformin combined with lifestyle interventions can prolong the onset of diabetes [3]. However, treatment with metformin may present toxic side effects, such as causing gastrointestinal discomfort and even lactic acidosis. Hyperlipidemia, also known as dyslipidemia, is a metabolic disease characterized by excess lipids in the blood plasma. A high-fat diet (HFD) or poloxamer-407 (P-407) can cause abnormal lipoprotein metabolism and lead to hyperlipidemia, which is characterized by elevated triglycerides (TG), total cholesterol (TC), low-density lipoprotein (LDL), and very low-density lipoprotein (VLDL), while the level of high-density lipoprotein (HDL) is decreased [4]. Among lipid-lowering drugs, statins and resins are mainly used to reduce TC and LDL, while niacin is used to reduce TG. The most common side effects of simvastatin are muscle pain and gastrointestinal symptoms [5]. Nonalcoholic fatty liver disease (NAFLD) refers to a metabolic disease in which the lipid content in the liver exceeds 5~10%, and its etiology has nothing to do with alcohol, drugs, and genetics [6]. The prevalence of NAFLD in ordinary adults is 10%~30%. Pioglitazone can be used to treat NAFLD, yet with possible side effects such as weight gain and hypoglycemia [7].

Due to the acceleration of population aging and changes in behavior and socioeconomics, metabolic diseases have become a serious public health problem. Therefore, there is an urgent need to seek safe and effective therapeutic drugs. Phytotherapy has been valued in diverse traditional cultures and it is believed that natural products are more economical and safer than chemical products [8]. Because of their therapeutic properties and safety, natural compounds from vegetables, fruits, or medicinal plants are widely used in the prevention or/and treatment of diseases. As a super family of phytochemicals, flavonoids extracted from natural plants are reported to have potential therapeutic value for metabolic diseases [9]. Quercetin is an important flavonoid compound found in many edible and medicinal plants. In recent years, quercetin has received widespread attention due to its good potential in the treatment of metabolic diseases. It has a variety of pharmacological activities, such as hypoglycemic, hypolipidemic, cardiovascular protecting, anti-inflammatory, anticancer, and hepatoprotective effects [10–12]. Some clinical trials have also reported its beneficial effects on type 2 diabetes mellitus (T2DM), hyperlipidemia, and NAFLD [13–15]. However, most of these reports are scattered and lack a systematic summary.

In this review, the natural sources and physicochemical properties of quercetin were introduced. Its absorption, metabolism, and bioavailability were also outlined. More importantly, we provide a timely and comprehensive overview of the pharmacological advances and clinical evidence of quercetin in the treatment of three metabolic diseases (diabetes, hyperlipidemia, and NAFLD). The information may provide an important reference for the future research and development of quercetin.

## 2. Methods

In this review, we performed a comprehensive literature search using some online databases (e.g., ISI Web of Science, PubMed, Science Direct, Google Scholar, and China National Knowledge Infrastructure) to obtain information on the effects of quercetin on three metabolic diseases. In the search process, the following keywords were used: “quercetin” and “metabolic diseases”, “diabetes”, “hyperlipidemia”, or “non-alcoholic fatty liver disease”. The references of all retrieved articles were also reviewed to identify other articles that may be missed using the above search terms. After the literature search, we screened the titles and abstracts of the extracted studies and further examined the full text of the included articles to confirm eligibility for inclusion in this review. Editorials, meeting abstracts, and studies with incorrect, incomplete, or unavailable data were excluded.

## 3. Natural Sources and Physicochemical Properties of Quercetin

### 3.1. Natural Sources

As one of the most abundant dietary flavonoids in the human diet to date, quercetin is widely distributed in vegetables and fruits. Xiao et al. corrected the previous misconception and what they believed is that quercetin intake accounted for about 60%-75% of total flavonols, not total dietary flavonoids [16]. The sources of quercetin differ in different countries and regions due to the diversity of regional environment, traditional culture, and other factors. In Chinese population, the main dietary sources of quercetin were green vegetables, tubers, tomatoes, oranges, apples, green tea, pears, sweet potato, and black tea [13]. In Japan, quercetin was mainly provided by green tea and onion in summer and winter. In the meanwhile, some summer vegetables, such as green pepper, asparagus, red lettuce, and tomato, were also good sources of quercetin [17]. In Australian adults, apples, black tea, grapes, lettuce, green beans, tomatoes, mature onions, green tea, coffee, and wine were the major sources of quercetin [18]. Häkkinen et al. reported that the main sources of quercetin were berry, apple, tea, and onion in Finland [19].  Table 1 lists the natural sources of quercetin [20–38].

Moreover, quercetin can also be obtained from traditional medicinal plants. It is widely distributed in the roots, leaf, and fruit of many medicinal plants, such as *Petroselinum crispum* (Mill.) Fuss (Apiaceae), *Nepeta cataria* L. (Labiatae), *Ginkgo biloba* L. (Ginkgoaceae), *Mentha canadensis* L. (Labiatae), *Polygonum orientale* L. (Polygonaceae), and *Crataegus pinnatifida* Bunge (Rosaceae). High-performance liquid chromatography method is commonly used to determine the content of quercetin in herbal medicines. The content of quercetin varies greatly in different plants, ranging from 0.02 mg/g to 63.60 mg/g. Among the reported plants, *P. orientale* has the highest concentration of quercetin (63.60 mg/g), followed by *G. biloba* (33.00 mg/g) and *M. canadensis* (10.80 mg/g).

### 3.2. Physical and Chemical Properties of Quercetin

**Figure 1:**
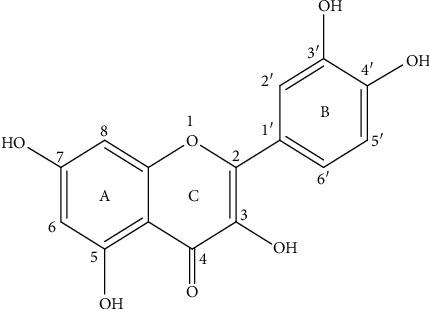
The chemical structure of quercetin.

Quercetin is a bright yellow needle-like crystal with a melting point of 313-314°C. Its molecular formula is C_15_H_10_O_7_ and molecular mass is 302.23. Quercetin is also known as 3,3′,4′,5,7-pentahydroxyflavone or 3,3′,4′,5,7-pentahydroxy-2-phenylchromen-4-one (Figure 1). The basic carbon chain of quercetin is C6-C3-C6, which consists of two benzene rings (A and B) and an oxygen-containing heterocycle (C). There is a hydroxyl group at the 3, 3′, 4′, 5, and 7 positions, respectively. The molecular structure of quercetin is known as the best configuration for scavenging free radicals and binding transition metal ions, so it is considered to have a potent antioxidant effect [39]. Chemical stability of quercetin is affected by many factors, such as pH, temperature, storage time, and the presence of oxygen and metal ions [40]. Quercetin is unstable in organic solutions with a pH greater than 7, which may be due to its central ring structure. Moreover, quercetin is a common flavonoid aglycone, so it is soluble in ethanol, methanol, and ethyl acetate, hardly soluble in petroleum ether, benzene, ether, and chloroform, and almost insoluble in water.

## 4. Bioavailability of Quercetin

The bioavailability of quercetin is closely related to its biological activity and health benefits. In view of the wide application of quercetin-containing products and the drug-forming potential of quercetin, its bioavailability should be carefully evaluated. Previous investigations have shown that quercetin has poor and highly variable bioavailability [41], which may be related to its pharmacokinetic properties. Absorption is an important factor affecting the oral bioavailability of quercetin. Pharmacokinetic studies suggest that quercetin has low absorption [41]. Chen et al. found that after oral administration, only 6.7% of quercetin was absorbed into the portal vein, while about 93% was lost in the intestine [42]. However, quercetin derivatives, especially its glycosides, glucuronide, or sulfate conjugates, are more easily absorbed than quercetin [42, 43]. In the intestine, quercetin could be degraded by some intestinal bacteria (e.g., *Eubacterium oxidoreducens*, *Clostridium orbiscindens*, and *Eubacterium ramulus*), with C ring fission and dehydroxylation to produce lower molecular weight phenolic compounds, which were easily absorbed [44]. Moreover, quercetin could be glucuronidated by uridine diphosphate glucuronosyltransferase and methylated by catechol-O-methyltransferase in the small intestine. Subsequently, they were transported to the liver through the portal circulation to be further metabolized, involving not only O-methylation and glucuronidation, but also sulfonylation by sulfotransferases [45]. Chen et al. found that approximately 93.3% of quercetin was metabolized in the intestine and only 3.1% was metabolized in the liver in rats [42]. The absorbed quercetin and its derivatives are mainly excreted into bile or urine and eventually eliminated in feces [40]. Quercetin bioavailability may be affected by its hepatic biliary excretion [41]. In addition, there are obvious individual differences in the bioavailability of quercetin, which is related to some endogenous (e.g., gender, age, and intestinal permeability) and exogenous factors (e.g., food matrix, dietary fat, and glycosylation of quercetin) [41, 46]. Besides, the duration and frequency of medication also affect the plasma concentration of quercetin. Some studies indicated that long-term or repeated supplement of quercetin could increase its bioavailability [40, 47]. The above findings suggest that a variety of factors can affect the oral bioavailability of quercetin. There is no doubt that improving the bioavailability of quercetin and its metabolites is expected to enhance its biological activity and health benefits.

## 5. Pharmacological Effects and Molecular Mechanisms of Quercetin against Three Metabolic Diseases

### 5.1. Diabetes

**Figure 2:**
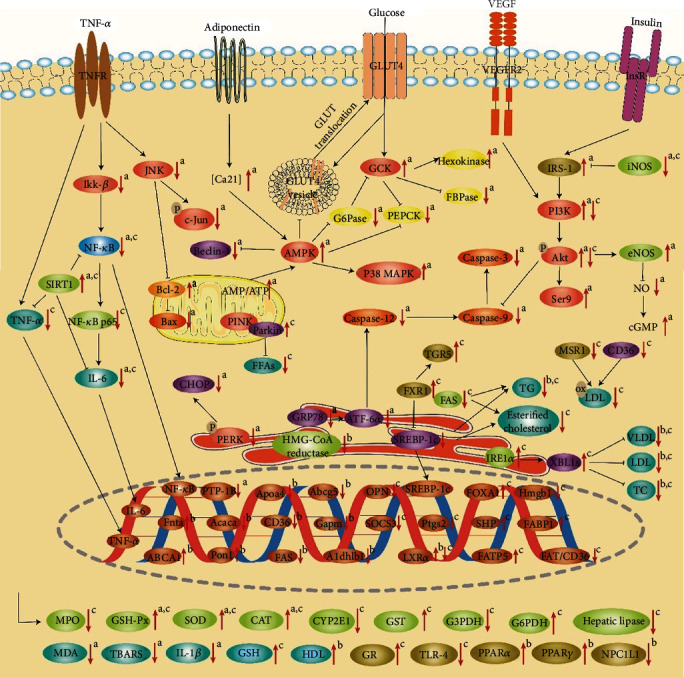
Quercetin can improve diabetes, hyperlipidemia, and NAFLD by modulating the marked targets. ↑ indicates increase and ↓ indicates decrease; → indicates stimulatory effect and ⊣ indicates inhibitory effect. In the upper right corner, a represents the effect of quercetin on diabetes, b represents the effect of quercetin on hyperlipidemia, and c represents the effect of quercetin on NAFLD. ABCA1: ATP-binding cassette transporter A1; Abcg5: ATP-binding cassette subfamily G member 5; Acaca: acetyl-coenzyme A carboxylase *α*; Akt: protein kinase B; Aldh1b1: aldehyde dehydrogenase 1 family member B1; AMPK: adenosine monophosphate-activated protein kinase; Apoa4: apolipoprotein A-IV; ATF-6*α*: activating transcriptional factor 6*α*; CAT: catalase; CD36: cluster of differentiation 36; cGMP: cyclic 3,5-guanosine monophosphate; CHOP: CCAAT/enhancer-binding protein homologous protein; CYP2E1: cytochrome P450 2E1; eNOS: endothelial nitric oxide synthase; FABP1: fatty acid-binding protein 1; FAS: fatty acid synthase; FAT/CD36: fatty acid translocase CD36; FATP5: fatty acid transport protein 5; FFAs: free fatty acids; FOXA1: forkhead box protein A1; FXR1: farnesoid X receptor 1; FAS: fatty acid synthase; FBPase: fructose-bisphosphatase; Fnta: farnesyltransferase CAAX box *α*; G6Pase: glucose-6-phosphate; G3PDH: glycerol-3-phosphate dehydrogenase; G6PDH: glucose-6-phosphate dehydrogenase; GCK: glucokinase; GLUT4: glucose transporter type 4; Gpam: glycerol-3-phosphate acyltransferase mitochondrial; GR: glutathione reductase; GRP78: glucose regulated protein 78; GSH: glutathione; GSH-Px: glutathione peroxidase; GST: glutathione-S-transferase; HDL: high-density lipoprotein; Hmgb1: high-mobility group box 1; HMG-CoA reductase: 3-hydroxy-3-methylglutaryl-CoA reductase; Ikk-*β*: inhibitor *κ*B kinase *β*; IL-1*β*: interleukin-1*β*; IL-6: interleukin-6; iNOS: inducible nitric oxide synthase; InsR: insulin receptor; IRE1*α*: inositol-requiring transmembrane kinase/endoribonuclease 1*α*; IRS-1: insulin receptor substrate-1; JNK: c-Jun-NH_2_ terminal kinase; LDL: low-density lipoprotein; LXR*α*: liver X receptor *α*; MDA: malondialdehyde; MPO: myeloperoxidase; MSR1: macrophage scavenger receptor 1; NF-*κ*B: nuclear factor-*κ*B; NO: nitric oxide; NPC1L1: Niemann-Pick C1-like 1; OPN: osteopontin; ox-LDL: oxidized low-density lipoprotein; P38 MAPK: P38 mitogen-activated protein kinases; PEPCK: phosphoenolpyruvate carboxylase; PERK: protein kinase-like endoplasmic reticulum kinase; PI3K: phosphatidyl inositol 3 kinase; Pon1: paraoxonase1; PPAR*α*: peroxisome proliferator-activated receptor *α*; PPAR*γ*: peroxisome proliferator-activated receptor *γ*; Ptgs2: cyclooxygenase-2; PTP-1B: phosphatase-1B; Ser9: phosphorylated glycogen synthase kinase 3*β*; SHP: small heterodimer partner; SIRT1: sirtuin 1; SOCS3: suppressor of cytokine signaling 3; SOD: superoxide dismutase; SREBP-1c: sterol regulatory element-binding protein-1c; TBARS: thiobarbituric acid-reactive substances; TC: total cholesterol; TG: triglyceride; TGR5: Takeda G protein-coupled receptor 5; TLR-4: Toll-like receptor-4; TNFR: tumor necrosis factor receptor; TNF-*α*: tumor necrosis factor-*α*; VEGF: vascular endothelial growth factor; VEGFR2: vascular endothelial growth factor receptor 2; VLDL: very low-density lipoprotein; XBP1s: X-box-binding protein 1.

Accumulating evidences indicate that quercetin has good antidiabetic effects. Quercetin may exert antidiabetic effects through various mechanisms, including promoting insulin secretion, improving insulin resistance, maintaining glucose homeostasis, and inhibiting inflammation, oxidative stress, and apoptosis. The main targets modulated by quercetin are shown in Figure 2.

#### 5.1.1. Quercetin Promotes Insulin Secretion

Healthy individual maintains a stable blood glucose level through basal insulin secretion, while diabetic hyperglycemia is the main symptom caused by insulin deficiency. Bhattacharya et al. found that quercetin had the potential to promote glucose-stimulated insulin secretion and insulin expression in INS-1E (insulin-secreting rat insulinoma) cells [48]. A study reported that quercetin could resist cholesterol-induced pancreatic *β* cell dysfunction, thereby maintaining glucose-stimulated insulin secretion and glycemic control [49]. Another study found that quercetin was able to dose-dependently improve insulin secretion and reduce blood glucose levels after 6 weeks of oral administration in diabetic rats [50]. In addition, quercetin could accelerate the recovery of *β* cells by increasing the expression of vascular endothelial growth factor (VEGF) and its receptor VEGFR2 in the pancreas of diabetic rats [50]. Youl et al. found that quercetin could significantly enhance the insulin secretion of INS-1 pancreatic *β* cells by specifically activating extracellular signal-regulated kinase 1/2 (ERK1/2) [51]. Moreover, Bardy et al., Zhuang et al., and Kittl et al. found that quercetin significantly stimulated insulin secretion by activating the intracellular Ca^2+^ signaling pathway [52–54].

#### 5.1.2. Quercetin Improves Insulin Resistance

El-Baky reported that quercetin could decrease insulin resistance evaluated by homeostasis model assessment (HOMA) in diabetic rats [55]. Shao et al. found that quercetin significantly reduced fasting glucose and increased insulin sensitivity index, causing significant changes in the insulin level of diabetic rats [56]. Gaballah et al. discovered that quercetin treatment could significantly improve insulin resistance in HFD and streptozotocin- (STZ-) induced type 2 diabetic rats through the alleviation of pancreatic endoplasmic reticulum (ER) stress and oxidative stress, as well as counteraction of inflammation and *β* cell death [57]. Chuang et al. found that quercetin attenuated the tumor necrosis factor-*α*- (TNF-*α*-) mediated insulin resistance in primary human adipocytes by blocking the serine phosphorylation of insulin receptor substrate-1 (IRS-1) and the expression of phosphatase- (PTP-) 1B gene [58]. In addition, quercetin affected the hypothalamic insulin signaling pathway by upregulating the phosphorylation of insulin receptors (InsRs) and protein kinase (PK) B, also known as Akt, thereby effectively improving insulin resistance in high fructose-induced rats [59]. Moreover, Dhanya et al. found that the adenosine monophosphate-activated protein kinase (AMPK) signaling pathway could help quercetin correct insulin resistance by bypassing the GLUT4 translocation of the insulin regulatory system in L6 myotubes [60].

#### 5.1.3. Quercetin Maintains Glucose Homeostasis

Diabetes patients often have disorders of glucose metabolism. Sandeep and Nandini found that quercetin treatment restored the impaired protein expression of key insulin signaling molecules, such as IRS-1 and phosphatidyl inositol 3 kinase (PI3K), thereby promoting insulin-mediated glucose uptake in the brain of STZ-induced diabetic rats [61]. In the liver of rats, quercetin induced AMPK activation, which reduced glucose production mainly by downregulating key glycogenic isoenzymes, such as glucose-6-phosphate (G6Pase) and phosphoenolpyruvate carboxylase (PEPCK) [62, 63]. Peng et al. found that quercetin significantly enhanced the expression of phosphorylated glycogen synthase kinase 3*β* (GSK-3*β*), namely, Ser9, and Akt phosphorylation promoted the expression of glucokinase (GCK) protein (known as hexokinase type IV) in the liver of diabetic rats so as to enhance the synthesis of hepatic glycogen and alleviate the glucose metabolism disorders [64]. The research of Eid et al. and Alam et al. shows that quercetin increases glucose uptake by stimulating the translocation of the glucose transporter type 4 (GLUT4) to the plasma membrane in murine skeletal muscle cells [62, 65]. Dhanya et al. and Eid et al. used PK inhibitors to demonstrate that quercetin exerted its role in ameliorating glucose uptake in L6 myotubes through the adenosine AMPK pathway and its downstream target, P38 mitogen-activated protein kinases (MAPK) [60, 66]. Peng et al. and Xiao et al. reported that quercetin could promote glucose processing in adipocytes and liver by regulating the activity of sirtuin 1 (SIRT1) [64, 67]. Alam et al. and Vessal et al. found that the activity of hexokinase was increased, while G6Pase and fructose-bisphosphatase (FBPase) activities were decreased in quercetin-treated diabetic mice and rats [65, 68]. These results indicated that quercetin could reduce gluconeogenesis and increase glycolysis, ultimately reducing hyperglycemia and altering glucose metabolism disorders [65, 68]. In addition, quercetin, as an inhibitor of carbohydrate hydrolases, such as pancreatic *α*-amylase and intestinal *α*-glucosidase, could slow down the hydrolysis of starch, thereby reducing the rate of glucose absorption and leading to a delayed rise in postprandial hyperglycemia [69, 70].

#### 5.1.4. Quercetin Inhibits Inflammation in Diabetic Animals

Quercetin has been reported to have obvious anti-inflammatory effects. Rifaai et al. found that inflammatory cell infiltration could be reduced by quercetin in pancreatic islets of STZ-induced diabetic rats [71]. Eitah et al. and Rivera et al. found that the anti-inflammatory effect of quercetin could be produced by increasing plasma adiponectin and reducing plasma nitrate plus nitrite (NOx) and TNF-*α* concentrations in diabetic rats [72, 73]. In primary human adipocytes, quercetin could reduce TNF-*α*-mediated inflammation [58]. It could directly inhibit the activation of ERK, c-Jun-NH_2_ terminal kinase (JNK), c-Jun, and nuclear factor-*κ*B (NF-*κ*B), which induced inflammatory gene expression and protein secretion, and indirectly activate peroxisome proliferator-activated receptor *γ* (PPAR*γ*) activity [58]. Similarly, quercetin significantly decreased the serum concentrations of some proinflammatory mediators, such as interleukin-6 (IL-6), in women with T2DM [74]. In another study, the levels of inhibitor *κ*B kinase *β* (Ikk-*β*), TNF-*α*, and IL-1*β* were decreased, and the expressions of Ikk-*β* and TNF-*α* protein were also reduced in the heart of diabetic rats after quercetin treatment [75].

#### 5.1.5. Quercetin Inhibits Oxidative Stress in Diabetic Animals

Oxidative stress has a potential role in the pathogenesis of diabetes. Fedosova et al. found that quercetin could reduce the oxidative function of polymorphonuclear neutrophils (PMN) in patients with non-insulin-dependent diabetes mellitus (NIDDM) [76]. Quercetin exerted its antioxidant effects by directly inhibiting the production of lipid peroxides, such as malondialdehyde (MDA) and thiobarbituric acid-reactive substances (TBARS), and indirectly promoting the production of endogenous antioxidants, such as antioxidant enzymes glutathione peroxidase (GSH-Px), superoxide dismutase (SOD), and catalase (CAT), in STZ-induced diabetic rats and db/db mice [55, 75, 77–85]. Dokumacioglu et al. and Chis et al. reported that quercetin could ameliorate oxidative stress in STZ-induced diabetic rats through scavenging oxygen free radicals [82, 84]. Iskender et al. found that quercetin inhibited oxidative damage by increasing SIRT1 level and decreasing NF-*κ*B level in STZ-induced diabetic rats [85]. Moreover, quercetin could prevent oxidative stress in liver tissues of STZ-induced diabetic rats by inhibiting the apoptosis process of target cells [86]. Quercetin could significantly reduce inducible nitric oxide synthase (iNOS) activity and nitric oxide (NO) concentration in the serum of diabetic mice [87]. In addition, quercetin could also protect the function and viability of INS-1 *β* cells from oxidative damage by enhancing ERK1/2 phosphorylation [51]. Lopez et al. found that quercetin might be helpful in reducing oxidative damage of pancreatic insulinoma *β* cells by regulating the polymerization tendency of human amylin, which is a pathological marker of T2DM [88].

#### 5.1.6. Quercetin Inhibits Apoptosis

Modern investigations have shown that quercetin can reduce apoptosis in diabetic animals. By using the terminal dUTP nick end-labeling (TUNEL) method, Kanter et al. found that quercetin ameliorated testicular cell apoptosis of rats induced by diabetes [89]. Quercetin could significantly decrease the levels of proapoptotic markers (caspase-3, caspase-9, and Bax), while increasing the level of antiapoptotic markers (Bcl-2) in diabetic rats and mice [75, 87, 90]. Lin et al. also reported that quercetin-rich guava juice lowered the expression of autophagy-related proteins (Beclin-1 and LC3-B) and a biomarker of pyroptosis (IL-1*β*) in the pancreas and kidney of T2DM rats [90]. In addition, quercetin was reported to reduce pancreatic *β* cells' apoptosis by increasing endothelial nitric oxide synthase (eNOS) and regulating the NO-cyclic 3′,5′-guanosine monophosphate (cGMP) signaling in endothelial cells [91].

Some studies have shown that quercetin can inhibit ER stress, which plays an important role in reducing apoptosis. Cai et al. reported that quercetin could inhibit ER stress to prevent glucosamine-induced RAW264.7 macrophages apoptosis [92]. Specifically, it decreased the expression of glucose regulated protein 78 (GRP78) and inhibited the activation of activating transcriptional factor 6*α* (ATF-6*α*) to reduce CCAAT/enhancer-binding protein (C/EBP) homologous protein (CHOP) expression and inhibit JNK phosphorylation and caspase-12 activation [92]. Another study found that quercetin treatment could also restore ER homeostasis by reducing the phosphorylation of protein kinase-like ER kinase (PERK) and the expression of caspase-3, thus reducing the apoptosis of human umbilical vein endothelial cells (HUVECs) [93].

### 5.2. Hyperlipidemia

Many investigations have shown that quercetin has good pharmacological activity on hyperlipidemia. It could reduce the levels of TG, TC, LDL, and VLDL, inhibit 3-hydroxy-3-methylglutaryl-CoA (HMG-CoA) reductase activity, and increase HDL levels in hyperlipidemic animals [14, 56, 94]. Sikder et al. found that quercetin could ameliorate HFD-induced dyslipidemia in Swiss albino mice [95]. In detail, quercetin treatment reduced serum TC, TG, and LDL levels by 30%, 34%, and 22%, respectively. Cholesterol and TG levels were also reduced by 32% and 21%, respectively, by quercetin treatment [95]. Similarly, Mariee et al. reported that quercetin administration for 3 weeks could significantly reduce the levels of serum TC (20%), liver TC (22%), liver TG (24%), and serum LDL cholesterol (LDL-c) (31%) and significantly increase serum HDL cholesterol (HDL-c) levels [96]. Juzwiak et al. studied the effect of quercetin on experimental hyperlipidemia in rabbits. The results demonstrated that quercetin taken for 12 weeks could effectively reduce serum TG and cholesterol levels elevated by high-fat diet [97]. A recent study indicated that pretreatment with quercetin by oral gavage for a period of 30 days decreased TC and increased HDL-c levels in hyperlipidemic rats induced by P-407 [98]. Moreover, quercetin could decrease plasma cholesterol and prevent the hypertrophy of the left ventricular in hypercholesterolemic mice [99]. Hu et al. also found that quercetin at 50 and 100 mg/kg could effectively improve fructose-induced dyslipidemia in rats. It significantly lowered the serum TG, TC, and VLDL levels in rats [100].

The mechanism of quercetin's antihyperlipidemic effect may involve multiple aspects (Figure 2). Quercetin could reduce high blood cholesterol levels by specifically inhibiting the absorption of intestinal cholesterol through reducing the expression of the epithelial cholesterol transporter Niemann-Pick C1-like 1 (NPC1L1) [101]. Jung et al. and Kobori et al. found that quercetin supplementation improved dyslipidemia through reducing oxidative stress, increasing PPAR*α* expression, and improving the expression of some genes related to lipid metabolism in mice, including farnesyltransferase CAAX box *α* (Fnta), paraoxonase 1 (Pon1), aldehyde dehydrogenase 1 family member B1 (Aldh1b1), ATP-binding cassette subfamily G member 5 (Abcg5), apolipoprotein A-IV (Apoa4), acetyl-coenzyme A carboxylase *α* (Acaca), fatty acid synthase (FAS), cluster of differentiation 36 (CD36), glycerol-3-phosphate acyltransferase mitochondrial (Gpam), and sterol regulatory element-binding protein-1c (SREBP-1c) [102, 103]. Another study indicated that quercetin might treat hyperlipidemia by promoting PPAR*γ* and liver X receptor *α* (LXR*α*) expressions to upregulate ATP-binding cassette transporter A1 (ABCA1) genes and increasing cholesterol efflux from THP-1 macrophages in human acute monocytic leukemia cells [104].

### 5.3. NAFLD

The pathogenesis of NAFLD involves multiple mechanisms, such as lipid accumulation, inflammation, and oxidative stress. Emerging evidence has shown that quercetin can effectively treat NAFLD by reducing lipid accumulation, antioxidation, and anti-inflammation and restoring disturbed metabolites and gut microbiota (Figure 2).

#### 5.3.1. Quercetin Ameliorates Hepatic Lipid Accumulation

A high-fat and high-sucrose (HFS) diet can induce NAFLD in male rats. Compared with the HFS diet group, quercetin was found to significantly reduce the content of TC and TG in the liver of NAFLD rats [105]. Meanwhile, the assays for hepatic enzymes involved in lipid metabolism showed that the activity of glucose-6-phosphate dehydrogenase (G6PDH) was significantly increased, while the activity of hepatic lipase and glycerol-3-phosphate dehydrogenase (G3PDH) was significantly decreased by quercetin. Moreover, in HFD-induced nonalcoholic steatohepatitis (NASH) in rats, the concentrations of lipids and lipoproteins, including free fatty acids (FFAs), free cholesterol, esterified cholesterol, TC, TG, LDL, and VLDL, were significantly reduced after 4 weeks of treatment with quercetin (20 mg/kg) [106]. It was reported that quercetin could reduce hepatic lipid accumulation, thereby improving HFD-induced NAFLD by promoting hepatic VLDL assembly and lipophagy via the inositol-requiring transmembrane kinase/endoribonuclease 1*α*/X-box-binding protein 1 (IRE1*α*/XBP1s) pathway. Specifically, compared with the HFD group, quercetin decreased hepatic TG content by 39% and TC by 28%, resulted in a 1.5-fold increase in hepatic VLDL, and upregulated the expression of spliced XBP1s [107]. Pisonero-Vaquero et al. found that quercetin reduced lipid accumulation by modulating lipid metabolism-related gene expression via the PI3K/Akt pathway inactivation in a diet-induced mouse model of NAFLD [108]. Specifically, it significantly downregulated the expression of two *de novo* lipogenesis genes including SREBP-1c and LXR*α* and a fatty acid uptake- and trafficking-related gene, fatty acid translocase CD36 (FAT/CD36). Moreover, quercetin was able to upregulate the expression of fatty acid transport protein 5 (FATP5), fatty acid-binding protein 1 (FABP1), forkhead box protein A1 (FOXA1), and small heterodimer partner (SHP) [108]. Similarly, Li et al. proved that quercetin could significantly improve hepatic lipid accumulation and decrease the levels of TG by suppressing two lipogenesis gene expression levels of SREBP-1c and FAS [109]. Recently, Liu et al. found that quercetin could prevent FFA-induced lipid accumulation *in vitro* and alleviate HFD-induced hepatic steatosis *in vivo* through increasing frataxin-mediated PTEN-induced putative kinase 1 (PINK1)/Parkin-dependent mitophagy [110]. In addition, HFD-induced NAFLD is usually accompanied by oxidized low-density lipoprotein (ox-LDL) deposition in the liver. Liu et al. found that quercetin administration for 24 weeks (100 mg/kg) could significantly alleviate HFD-induced liver damage and reduce hepatic cholesterol and ox-LDL levels by improving the autophagy lysosomal signaling pathway and inhibiting the expression of scavenger receptors, including macrophage scavenger receptor 1 (MSR1) and CD36 [111]. Recently, a study showed that quercetin could reduce liver lipid accumulation in T2DM-induced NAFLD by activating the farnesoid X receptor 1/Takeda G protein-coupled receptor 5 (FXR1/TGR5) signaling pathway [112].

#### 5.3.2. Quercetin Alleviates Inflammation and Oxidative Stress in Hepatitis Animals

NASH is the inflammatory subtype of NAFLD. A study reported that quercetin treatment (20 mg/kg) showed a protective effect against HFD-induced NASH by reducing the levels of inflammatory markers TNF-*α* and myeloperoxidase (MPO) [113]. Similarly, Ying et al. found that quercetin could reduce serum levels of proinflammatory cytokines TNF-*α* and IL-6 by upregulating SIRT1 and downregulating iNOS and NF-*κ*B p65 in the NASH gerbils induced by a high-fat diet [114]. Moreover, Marcolin et al. reported that quercetin (50 mg/kg) could ameliorate inflammation and fibrosis in NASH mice by attenuating various proinflammatory and profibrotic gene pathways [115]. Specifically, it could significantly reduce the liver mRNA levels of IL-6, TNF-*α*, high-mobility group box 1 (Hmgb1), and cyclooxygenase-2 (Ptgs2), and the liver protein concentration of Toll-like receptor-4 (TLR-4).

In addition, a study reported that quercetin exhibited significant antioxidant property in experimental NASH rats [116]. Nonenzymatic antioxidant glutathione (GSH) and several antioxidant enzymes including CAT, SOD, GSH-Px, glutathione reductase (GR), and glutathione-S-transferase (GST) were significantly increased by quercetin (20 mg/kg). Similarly, Surapaneni et al. reported that quercetin treatment could significantly reduce the level of prooxidant enzyme Cytochrome P450 2E1 (CYP2E1) in the liver of NASH rats, thereby improving CYP2E-mediated oxidative stress [117]. Another study found that quercetin could attenuate lipoperoxidation by regulating inflammatory and oxidative/nitrosative stress-related genes in a diet-induced mouse model of NAFLD [108]. Specifically, it effectively reduced the mRNA levels of TNF-*α*, iNOS, osteopontin (OPN), and suppressor of cytokine signaling (SOCS) 3.

#### 5.3.3. Quercetin Regulates Serum Metabolites and Gut Microbiota in NAFLD Animals

Metabolomics is a common approach to study the pharmacological activity and molecular mechanism of natural products [118]. Recently, Xu et al. investigated the effects and mechanisms of quercetin on HFD-induced NAFLD using nontargeted metabolomics technology [119]. The results indicated that quercetin showed good hepatoprotective activity in 30-day NAFLD rats, and the underlying mechanisms might be related to its regulation of 2 inflammation-related metabolites (arachidonic acid and 12(S)-HPETE), 5 fatty acid-related metabolites (eicosapentaenoic acid, adrenic acid, oleic acid, docosahexaenoic acid, and palmitic acid), 1 oxidative stress-related metabolite (2-hydroxybutyric acid), and 5 other differential metabolites (*α*-dimorphecolic acid, citric acid, 15(S)-hydroxyeicosatrienoic acid, chenodeoxycholic acid glycine conjugate, and 9,10,13-trihydroxy-11-octadecenoic acid) [119].

In recent years, many studies have shown that gut microbiota is involved in the progression of NAFLD [120–122]. Porras et al. found that the protective effect of quercetin on NAFLD is also mediated by modulating gut microbiota disorders and related gut-liver axis [123]. Specifically, supplementation of quercetin for 16 weeks could significantly reduce the *Firmicutes*/*Bacteroidetes* ratio and the relative percentage of *Proteobacteria* phylum, and increase the total bacteria concentration in HFD-induced NAFLD mice. At the genus level, quercetin was found to considerably reduce the number of reads of *Desulfovibrio* and *Helicobacter*, and significantly increase the relative abundance of *Flavobacterium*, *Allobaculum*, and *Sutterella*. Moreover, this study also proved that quercetin could increase the concentration of beneficial metabolites derived from gut microbiota (acetate, propionate, and butyrate), restore the gut barrier function, and improve endotoxemia, gut-liver axis activation, and subsequent inflammatory gene overexpression in NAFLD mice [123]. Subsequently, they further studied the protective role of quercetin against NAFLD by gut microbiota transplantation. The results showed that gut microbiota transplantation from the HFD-nonresponder donor and the HFD-fed donor with the highest response to quercetin resulted in a protective phenotype against the development of NAFLD [124]. Recently, they revealed that this protective phenotype against NAFLD was associated with the increase of gut *Desulfovibrio* and *Oscillospira*, the decrease of gut *Bacteroides* and *Oribacterium*, the increase of secondary bile acids (BAs) in plasma and feces, the induction of hepatic BA transporters, and the inhibition of hepatic lipogenic and BA synthesis genes [125]. The above findings suggest that quercetin has good prebiotic capacity, and regulation of gut microbiota is one of the important mechanisms of quercetin in the treatment of NAFLD.

## 6. Clinical Evidence of Quercetin in Treating the Three Metabolic Diseases

Although there is a lot of *in vitro* and *in vivo* evidence that quercetin could be a promising therapeutic agent for diabetes, hyperlipidemia, and NAFLD, clinical trials must confirm its effectiveness in humans.  Table 2 summarizes the published clinical trials of quercetin in the treatment of the three diseases.

### 6.1. Diabetes

Several cohort studies have estimated the association between daily quercetin intake and the onset of diabetes. Yao et al. conducted a large-scale cross-sectional study on 14711 Chinese adults [13]. It was found that the daily intake of quercetin in Chinese adults was 20.9 ± 2.32 mg, and the intake of quercetin was significantly negatively correlated with the prevalence of T2DM. Another prospective study conducted a survey on 10054 Finnish citizens [126]. The daily intake of quercetin was 3.3 ± 2.4 mg, and a total of 526 cases of T2DM occurred during the follow-up period. More importantly, they found that a lower risk of T2DM tended to be associated with higher intake of quercetin and myricetin. Moreover, a randomized, double-blind, placebo-controlled crossover trial of 37 apparently healthy, nonsmoking adults found that supplementation with quercetin could significantly reduce the plasma concentration of methylglyoxal, a compound associated with diabetes and its complications [127]. These results indicate that quercetin may have beneficial effects in improving diabetes.

Currently, a small number of clinical trials have been implemented to study the impacts of quercetin on human subjects with diabetes and related disorders. A randomized blinded crossover study was designed to evaluate the beneficial effect of quercetin on postprandial hyperglycemia challenged with carbohydrates load in T2DM patients. The results showed that a single oral dose of quercetin (400 mg) could effectively suppress postprandial hyperglycemia in T2DM patients after maltose loading [128]. Zahedi et al. conducted a 10-week double-blind randomized clinical trial to evaluate the effects of quercetin on blood lipids, blood pressure, and inflammatory biomarkers in patients with T2DM [74]. 72 women with T2DM were divided into quercetin group and placebo group. Quercetin was given to participants at a dose of 500 mg per day. Compared with placebo, quercetin administration significantly reduced the patient's systolic blood pressure. Moreover, quercetin could significantly reduce serum HDL-c, TNF-*α*, and IL-6 levels, but there is no significant difference compared with the placebo group. In addition, an 8-week randomized clinical trial was conducted to study the effects of quercetin on glycemic control, oxidative stress, lipid profile, and insulin resistance in T2DM [129]. The results showed that oral quercetin (250 mg/day) for 8 weeks could significantly improve the antioxidant status of patients with T2DM. However, it did not significantly change the fasting blood glucose, serum insulin, and glycosylated hemoglobin (HbA1c) levels, and lipid profile in patients with T2DM. The difference between the results of these clinical trials may be due to the different doses of quercetin utilized and the duration of intervention. Recently, a systematic review and meta-analysis was performed to determine the effect of quercetin on glycemic control among patients with metabolic disorders [130]. This study concluded that quercetin can significantly reduce fasting plasma glucose levels when the dose is ≥500 mg/day and the duration is ≥8 weeks. Therefore, the dose and duration of intervention are the key factors that determine whether quercetin can effectively treat diabetes.

### 6.2. Hyperlipidemia

A double-blind randomized clinical trial was conducted to determine the effects of regular consumption of quercetin on blood lipid levels among asymptomatic persons with dyslipidemia. The results showed that giving quercetin for two months could decrease the levels of cholesterol, TG, and LDL, and increase HDL levels [14]. Moreover, Lu et al. studied the hypocholesterolemic effect of quercetin-rich onion juice on healthy mild hypercholesterolemia [131]. 24 subjects were divided into two groups, and they consumed 100 mL of onion juice or placebo every day. After 8 weeks of intervention, quercetin-rich onion juice significantly decreased waist circumference, TC, LDL-c, and LDL-c/HDL-c levels, elevated total antioxidation capacity, and prolonged the lag time of LDL oxidation. Although these two studies indicate that quercetin may have a positive effect on blood lipid levels, a meta-analysis of randomized controlled trials has yielded mixed results. The meta-analysis concluded that quercetin does not have any clinically relevant effects on plasma lipids, except for a significant reduction in TG at doses above 50 mg/day [9]. Moreover, the impact of quercetin on TG levels was found to be significantly associated with its dosage and duration of supplementation. Therefore, due to the small number of clinical trials and the limited sample size, the effectiveness of quercetin on hyperlipidemia cannot be reached with a definite conclusion, although certain trends can be observed. More clinical studies with larger sample sizes and longer trial durations are needed to verify its effectiveness against hyperlipidemia in human subjects.

### 6.3. NAFLD

So far, there are few clinical studies on the therapeutic effect of quercetin on NAFLD. Prysyazhnyuk and Voloshyn studied the effects of quercetin on blood biochemical indicators and pro- and anti-inflammatory cytokines in patients with NAFLD [15]. After 2 weeks of quercetin combined with basic treatment, the activities of aspartate aminotransferase (AST), alanine aminotransferase (ALT), and gamma-glutamyl transferase (GGT) were significantly reduced by 37.2%, 50.4%, and 89.9%, respectively. Moreover, the levels of TC, TG, and TNF-*α* significantly decreased by 16.7%, 33.3%, and 39.8%, respectively. These results suggest that quercetin has potential therapeutic value for NAFLD. Oxidative stress plays a key role in the pathogenesis of NAFLD and can also mediate the destruction and death of red blood cell (RBC). Recently, a randomized, double-blind, placebo-controlled trial was conducted to evaluate the effect of quercetin supplementation on hematological parameters in patients with NAFLD [132]. Specifically, 90 patients were supplemented with quercetin or placebo capsules twice a day for 12 weeks. Compared with the placebo group, the quercetin treatment group significantly increased RBC levels, but decreased the mean corpuscular volume, mean corpuscular hemoglobin, and ferritin levels.

As noted, the current clinical trials show that quercetin has a beneficial effect on some biomarkers associated with NAFLD. However, clinical studies on the effects of quercetin in NAFLD remain scarce. The number of patients enrolled in these trials is also small. Therefore, more high-quality clinical trials are needed to better understand the efficacy of quercetin on NAFLD in a wide range of populations.

## 7. Conclusions and Future Perspectives

Quercetin is a widely distributed natural flavonol compound. It can be obtained not only from daily vegetables and fruits (e.g., onions and apples) but also from some common medicinal plants (e.g., *G. biloba* and *P. orientale*). In recent years, quercetin has received widespread attention due to its exciting pharmacological potential and health benefits. In this review, we comprehensively summarized the pharmacological effects and molecular mechanisms of quercetin in the treatment of three metabolic diseases (diabetes, hyperlipidemia, and NAFLD). The information can provide useful references for future preclinical and clinical studies of quercetin.

Previous studies have shown that the oral bioavailability of quercetin is low, which will limit its therapeutic application. Therefore, an important issue that needs to be resolved in future research is to improve the bioavailability of quercetin. In recent years, nanotechnology has developed rapidly. Nanoformulations provide a promising strategy to solve the problems related to the bioavailability of quercetin [133]. Singh et al. found that quercetin-loaded nanomicelles could improve its bioavailability and antidiabetic activity [134]. Moreover, novel delivery forms (e.g., Quercetin Phytosome®) can also enhance the bioavailability of quercetin. Riva et al. reported that Quercetin Phytosome® did not affect the metabolic control of diabetic patients treated with metformin, which supports the application of quercetin in diabetic patients with no effect on conventional metformin treatment [135]. Besides, as mentioned previously, quercetin metabolites are more easily absorbed than quercetin. Therefore, additional research is needed to determine whether the health benefits of quercetin are directly or indirectly mediated by its metabolites.

Accumulating evidences show that quercetin has beneficial effects on these three metabolic diseases through various mechanisms. It can improve diabetes by promoting insulin secretion, improving insulin resistance, regulating glucose homeostasis, and inhibiting inflammation, oxidative stress, and apoptosis. The therapeutic mechanisms of quercetin on hyperlipidemia also involve multiple aspects. It can inhibit the absorption of intestinal cholesterol, reduce oxidative stress, downregulate the expression of some adipogenic genes (e.g., FAS and SREBP-1c), and promote the expression of PPAR*α*, PPAR*γ*, and LXR*α*. In addition, the therapeutic effect of quercetin on NAFLD can be attributed to its ability to ameliorate liver lipid accumulation, alleviate inflammation and oxidative stress, and regulate serum metabolic disorders.

In recent years, monotargeted drugs have been found to have limitations in the overall clinical outcome of patients, because diseases are usually caused by disturbances in multiple signaling pathways [136]. Moreover, monotargeted drugs are prone to drug resistance and side effects. Therefore, new drugs may need to undergo a shift from single target therapy to multitarget therapy. Multitargeted drugs may be less potent, but they can act on multiple targets to improve various symptoms of the disease [136]. As shown in Figure 2, quercetin can regulate a variety of signaling pathways, so it has multitarget therapeutic effects on the three metabolic diseases. However, some targets have only been proven at the cellular level or in an animal experiment. Therefore, more experiments are needed to confirm these targets. In future research, it is also necessary to identify the key target of quercetin in the treatment of these metabolic diseases by using gene knockout or gene silencing technology.

Many studies have shown that gut microbiota may be a potential therapeutic target for metabolic diseases [137]. Gut microbiota plays an important role in the treatment of diseases with herbal medicine through complex interactions [138]. Herbal chemicals closely affect gut microbiota composition. Reciprocally, the gut microbiota can biologically convert herbal chemicals into active metabolites. Therefore, regulating the gut microbiota or being metabolized by the gut microbiota may be the common ground for the therapeutic effect of quercetin on diabetes, hyperlipidemia, and NAFLD. As mentioned earlier, the regulation of gut microbiota has been proven to be one of the important mechanisms of quercetin in the treatment of NAFLD [123–125]. However, there are scarce studies on the role of gut microbiota in the treatment of diabetes and hyperlipidemia with quercetin. Only one study reported that quercetin improved STZ-induced diabetic peripheral neuropathy in rats by decreasing four pathogenic species and enriching two prebiotic species [139]. Therefore, the relationship between the effects of quercetin on these metabolic diseases and the gut microbiota remains to be elucidated. Moreover, the identification of specific bacterial strains that may contribute to the therapeutic effect of quercetin on NAFLD still needs further research.

Up to now, clinical studies on the effects of quercetin in diabetes, hyperlipidemia, and NAFLD remain scarce. The beneficial effects of quercetin on these three diseases have been observed in some human clinical trials. However, some studies have also reported inconsistent or conflicting results, especially for diabetes. The dosage and duration of treatment may be the key factors affecting the clinical effectiveness of quercetin. Therefore, definitive conclusions cannot be drawn until more clinical data are obtained. Overall, although many animal studies have reported the therapeutic effects of quercetin on the three metabolic diseases, its clinical application in humans still has a long way to go. More clinical trials with appropriate design are needed to verify the true impact of quercetin treatment on patients with diabetes, hyperlipidemia, and NAFLD. Besides, additional researches are necessary to further evaluate the dose-response effect of quercetin on these three diseases. The completion of these studies is expected to provide a scientific basis for the therapeutic potential of quercetin in human subjects. As we gain a deeper understanding of the health benefits of quercetin, its therapeutic value for metabolic diseases may become more apparent and widely accepted.

## Figures and Tables

**Table 1:** Natural sources of quercetin.

Category	Family	Sources	Used part	Content of quercetin (mg/g)	References
Diet	Ericaceae	Bog whortleberry (*Vaccinium uliginosum* L.)	Fruit	0.158	[19]
		Lingonberry (*Vaccinium vitis-idaea* L.)		0.146	
		Cranberry (*Vaccinium oxycoccos* Linnaeus)		0.12	
	Apiaceae	Parsley (*Petroselinum crispum* (Mill.) Fuss)	Herb	4.20	[20]
		Coriander (*Coriandrum sativum* L.)		6.10	
	Poaceae	Lemon grass (*Cymbopogon citratus* (DC.) Stapf)		0.12	
	Lamiaceae	Mint (*Mentha canadensis* Linnaeus)		10.80	
	Amaryllidaceae	Onion (*Allium cepa* L.)	Bulb	0.054-0.286	[21]
	Rosaceae	Apple (*Malus domestica* (Suckow) Borkh.)	Fruit	0.047	[22]
		Cherry (*Cerasus pseudocerasus* (Lindl.) G. Don)		0.026	
	Fabaceae	Caper (*Tamarindus indica* L.)		2.34	
	Brassicaceae	Broccoli (*Brassica oleracea* var. *botrytis* Linnaeus)	Flower	0.025	
	Solanaceae	Hot pepper (*Capsicum annuum* L.)	Fruit	0.177-0.507	
		Tomato (*Lycopersicon esculentum* Miller)		0.046	
	Asteraceae	Red lettuce (*Lactuca sativa* var. *ramosa* Hort.)	Leaves	0.403	
	Asparagaceae	Asparagus (*Asparagus schoberioides* Kunth)	Algae	0.076	
	Vitaceae	Grapes (*Vitis vinifera* L.)	Fruit	0.014	
	Theaceae	Black tea (*Camellia sinensis* (L.) O. Ktze.)	Leaves	2.05-2.556	[23]

Medicinal plants	Polygonaceae	*Polygonum orientale* L.	Fruit	63.60	[24]
	Rosaceae	*Crataegus pinnatifida* Bunge	Leaves and fruit	Leaves: 9.31; fruit: 13.27	[25, 26]
	Fabaceae	*Sophora japonica* L.	Bud and flower	Bud: 5.68; flower: 5.20	[27, 28]
	Araliaceae	*Acanthopanax senticosus* (Rupr. et Maxim.) Harms	Root	1.40	[28]
	Ginkgoaceae	*Ginkgo biloba* L.	Leaves	33.0	
	Myrtaceae	*Psidium guajava* L.	Leaves	3.62	[29]
	Cucurbitaceae	*Momordica charantia* L.	Fruit	1.44	
	Aquifoliaceae	*Ilex kudingcha* C. J. Tseng	Leaves	2.82	
	Polygonaceae	*Polygonum perfoliatum* (L.)	Herb	3.07	[30]
	Phyllanthaceae	*Phyllanthus emblica* L.	Fruit	0.071	[31]
	Convolvulaceae	*Cuscuta chinensis* Lam.	Seed	0.26	[32]
	Myrtaceae	*Baeckea frutescens* L.	Leaves	1.10	[33]
	Loranthaceae	*Taxillus chinensis* (DC.) Danser	Stem with leaves	0.53	[34]
	Ranunculaceae	*Clematis brevicaudata* DC.	Herb	1.51	[35]
	Lamiaceae	*Dracocephalum moldavica* L.	Root	4.48	[36]
	Euphorbiaceae	*Euphorbia helioscopia* L.	Leaves, stem, and root	Leaves: 1.42; stem: 0.021; root: 0.044	[37]
	Brassicaceae	*Brassica rapa* L.	Root	0.20	[38]

**Table 2:** Published clinical trials of quercetin in the treatment of T2DM, hyperlipidemia, and NAFLD.

Disease	Sample	Intervention measure	Dose of quercetin	Duration	Outcome	References
		(Test/control group)				
T2DM	72 female patients aged 35-55 years	Quercetin/placebo	500 mg, qd	10 weeks	Systolic blood pressure↓, HDL-c↓, IL-6↓, TNF-*α*↓	[74]
T2DM	47 patients (31 women and 16 men) aged 30-60 years	Quercetin/placebo	250 mg, qd	8 weeks	TAC↑, oxidized LDL↓, fasting blood glucose^∗^, insulin^∗^, HbA1c^∗^	[129]
T2DM	24 patients	Quercetin/placebo	400 mg, once	30 minutes	Postprandial hyperglycemia↓	[128]
Hyperlipidemia	400 patients	Quercetin/nothing	No report	2 months	Cholesterol↓, TG↓, LDL↓, HDL↑	[14]
Hypercholesterolemia	24 patients (10 men and 14 women)	Quercetin-rich onion juice/placebo	100 mL, qd	8 weeks	TC↓, LDL-c↓, LDL-c/HDL-c↓ Thiols↑, TBARS↓, lag time of LDL oxidation↑	[131]
NAFLD	71 patients and 45 healthy individuals	Quercetin combining with basic treatment/basic treatment/nothing	40 mg, tid	14-16 days	AST↓, ALT↓, GGT↓, TC↓, TG↓, TNF-*α*↓	[15]
NAFLD	90 patients aged 18-65 years	Quercetin/placebo	250 mg, bid	12 weeks	RBC↑, MCV↓, MCH↓, ferritin↓	[132]

↑ means increase, ↓ means decrease, and ∗ means no obvious change. ALT: alanine aminotransferase; AST: aspartate aminotransferase; qd: quaque die; bid: bis in die; tid: ter in die; GGT: *γ*-glutamyl transferase; HDL: high-density lipoprotein; HbA1c: glycosylated hemoglobin; IL-6: interleukin-6; LDL: low-density lipoprotein; MCH: mean corpuscular hemoglobin; MCV: mean corpuscular volume; NAFLD: nonalcoholic fatty liver disease; RBC: red blood cell; T2DM: type 2 diabetes mellitus; TAC: total antioxidant capacity; TBARS: thiobarbituric acid reactive substances; TC: total cholesterol; TG: triglyceride; TNF-*α*: tumor necrosis factor-*α*.

## Data Availability

No data were used to support this study.
